# Associations among health-promoting lifestyle, self-care agency and health-related quality of life in Bai older adults with hypertension in Yunnan China

**DOI:** 10.1186/s12877-022-03608-0

**Published:** 2022-12-07

**Authors:** Mengqi Du, Hongqian Kong, Lingyun Ran, Yuanyuan Ran, Leixao Bai, Yongxue Du, Hongxue Guan, Yong Dong, Ying Zhao

**Affiliations:** 1 Teacher’s College of Columbia University, New York, NY USA; 2 Disease Control and Prevention Center, Yunnan Qujing, China; 3 Nursing School of Kunming Medical University, Kunming, Yunnan China; 4 Hunan University of Finance and Economics, Changsha, China; 5 Disease Control and Prevention Center, Yunnan Lijiang, China

**Keywords:** Health-promoting lifestyle, Self-care agency, Health-related quality of life, Elderly, Bai ethnic minority

## Abstract

**Background:**

Previous Chinese studies focused on the prevalence and influential factors of hypertension; however, little is known about their self-care literacy and quality of life among the Bai older adults with hypertension. The purpose of this research was to explore the associations among health-promoting lifestyle, self-care agency, and health-related quality of life in Bai ethnic older patients with hypertension, as well as the related factors of hypertension self-care abilities.

**Methods:**

A total of 472 Bai ethnic hypertension older adults aged 60 and above were enrolled in this study voluntarily from 5 rural communities of the Bai ethnic group. The Exercise of Self-Care Agency Scale (ESCAS) was employed to assess the Self-care ability of hypertension for the subjects, the Health-promoting lifestyle profile II(HPLP-II) was utilized to evaluate the health behavior, and MOS 36-Item Short Form Health Survey (SF-36) was chosen to assess the HRQOL for the studying population. All descriptive analyses, including demographic characteristics, socio-economic status, and clinical characteristics were stratified by Bai hypertensive elderly. Pearson correlation analysis model was used to examine the associations among health-promoting lifestyle, self-care agency, and health-related quality of life in Bai ethnic elderly with hypertension.

**Results:**

The HPLP-II, ESCA, and of HRQOL levels of the subjects were low, and the mean HPLP and ESCA scores had no significant statistical variance among different age groups. Significant statistical differences were found in Bai elderly subjects in the domain of PF and PH as well as the overall score in SF-36(all *P*< 0.01), 60–64 year group had the highest score of the above three domains in SF-36 than other age groups. The SF-36 scores were positively associated with HPLP and ESCA levels.

**Conclusion:**

The HPLP-II, ESCA, and of HRQOL levels of the Bai subjects were poor in the Bai ethnic hypertensive elderly. The HRQOL scores of subjects were positively connected with HPLP-II and ESCA abilities. More attention should be paid to lifestyle, healthy behaviors, and self-care abilities improvements to enhance the better HRQOL of Bai minority older adults with hypertension.

## Background

Globally, 1.13 billion people were suffering from hypertension in 2015. However, only 25% of male hypertensive patients as well as 20% of female hypertensive were under control at the same time. An estimated 226 million hypertension patients in China accounted for more than 50% of hypertensive patients in East, Southeast, and South Asia [1]. The seventh national population census the older population who is aged 60 and above accounted for 18.7% of the whole population, which indicated the aging society is deepening in China [2]. It is reported that the standardized prevalence of hypertension in Chinese adults aged 18 and above was 23.2% from 2012 to 2015, meanwhile, the prevalence of hypertension in males was higher than that of females [3]. Yunnan, one of the most undeveloped provinces in China, has got 23.8% prevalence of hypertension among residents aged 18 and above in 2018 [4]. The prevalence of hypertension among older adults in Yunnan was not calculated until now.

Bai ethnicity is the 3rd largest ethnic group in Yunnan, which mainly inhabits Dali autonomous prefecture [5]. Previous research on hypertension of Bai people revealed that hypertension incidence in rural Bai people aged 50 and above was 41.7%, while 41.5% for male residents and 41.9% for female residents. Furthermore, only 7.5% of hypertension patients met the hypertension control standard [6]. There were only several researches identified the influential factors related to hypertension in Bai adult residents, furthermore, studies on rural older hypertensive patients were even rarer. Smoking, drinking, overweight, education level, marriage status, sleeping quality, medication adherence, dyslipidemia, and lack of physical exercises were considered important indicators to identify hypertension in Bai adult residents [7, 8].

HRQOL is an assessment of health state based on modern concept of healthcare, which reflects the physical, psychological, social and emotional well beings of individuals [9]. Previous researches strongly demonstrated that hypertension self-management had a significant positive effect on hypertension control, as well as HRQOL [10]. Self-care is an essential factor in the management of chronic diseases and is defined as a process of maintaining health through health-promoting practices and disease management [11, 12]. Hypertension self-care composes of medication adherence, limitation of sodium and fat diet, no smoking, alcohol drinking restriction, weight reduction, self-monitoring blood pressure, regular follow-up check-up, physical activity engagement, stress adjustment, and so on [13, 14]. Hypertension self-care practices may be affected by a multitude of factors such as education, hypertension self-care knowledge, social support, illness duration, age, health literacy, etc. [15, 16], which components are involved in the concept of health-promoting lifestyle. Previous Chinese studies focused on the prevalence and influential factors of hypertension; however, little is known about their self-care literacy and quality of life among the Bai older adults with hypertension. Therefore, the current study aimed to reveal their health-promoting lifestyle, self-care agency, health-related quality of life levels, and the associations among them.

## Methods

### Sampling collection

In total, 472 Bai ethnic elderly patients with hypertension, aged 60 years old or above, from 5 Bai rural communities of Yunnan province were recruited in this study using a multi-stage stratified sampling method and were face-to-face interviewed by trained researchers from Kunming Medical University of Yunnan province who could speak both Mandarin and Bai language. Inclusion criteria were as follow: Bai elderly aged 60 and above; hypertension diagnosis more than 6 months; at least one antihypertensive medication for the past 6 months; the mental ability to answer the interview questionnaire. The Bai ethnic hypertensive patients who did not take antihypertensive medication, or were diagnosed with hypertension less than 6 months, or live with serious hypertension complications such as stroke, paralysis were excluded from this research. The interviews were a series of questions and scales, for example, age, education level, course of hypertension, family history of hypertension, health-promoting lifestyle profile (HPLP), the exercise of self-care Agency scale (ESCA), and the MOS item short-form health survey (SF-36).

### Ethical considerations

The protocol of this study was approved by Kunming Medical University and the local government from the beginning of the research. Written and verbal informed consent were obtained from all participants who were volunteering before this survey. All personal information of the participants was assured to be kept confidential.

### Measurement instruments

Health-promoting lifestyle profile II(HPLP-II) is a widely used instrument to evaluate an individual’s health behaviors and lifestyles, which showed good validation and reliability in multiple studies [17, 18]. It composes 6 aspects of health promotion behaviors and lifestyles, namely, health responsibility (9 items), physical activity (8 items), nutrition (9 items), spiritual growth (9 items), interpersonal relationships (9 items), and stress management (9 items). Each item is answered with four choices: never, sometimes, often, with a score of 1, 2, 3, and 4. The total score of health promotion lifestyle is divided into 4 grades, which are “Low”(52–90 points), “Average” (91–129 points), “Good” (130–169 points), and “Excellent” (170–208 points) respectively [19]. The internal consistency reliability of HPLP -II in this research is 0.939, and the Cronbach’s α coefficient of each subcategory of HPLP -II were 0.900, 0.860, 0.857, 0.901, 0.930, 0.942, respectively.

The Exercise of Self-Care Agency Scale (ESCAS) was chosen to measure the Self-care ability of hypertension in the Bai ethnic elderly. The ESCAS has been proven to have high reliability and validity in some international studies [20]. The scale consists of 43 items that assess the extent of self-care abilities of individuals on a 5-point Likert scale ranging from 1 = completely un-similar to me, while 5 = completely similar to me. The ESCA is divided into 3 levels according to the total score (172 points) of the scale: a high level indicating more than 66% of the total score, 33-66% of the total score for a medium level, a low level less than 33% of the total. Subjects with scores less than 33.3% (≤ 56), 33.3–66.6% (57–114), and more than 66.6% (≥ 115) of the total score are considered with low, moderate, and high self-care agency, respectively. The higher score indicates better self-care agency. The Cronbach’s α coefficient of the ESCA scale in this study was 0.891.

Health-related quality of life is an individual’s subjective perception of physical, psychological, social and emotional well beings and a comprehensive indicator that comprehensively reflects the individual’s health status. The SF-36 scale is widely used to measure the quality of life in the general population. The SF-36 scale consists of eight areas: physical functioning (PF), role limitations relating to physical health (RP), bodily pain (BP), general health perception (GH), vitality (VT), social functioning (SF), role limitation relating to emotional health (RE) and mental health (MH). The Physical Component Summary includes PF, RP, BP, GH and Mental Component Summary contains VT, SF, RE, and MH. The score of each domain ranges from 0 to 100, with higher scores indicating a better-perceived quality of life [21]. According to the grading cut-off point of the Chinese version of the SF-36 scale recommended by Zhang Lei as the classification standard, the total score is greater than 117, indicating that the research object is in good condition; and the total score is in the range from 72 to 117, indicating that the research object is in moderate condition; and the total score below 72 indicates that the research subjects are in poor condition [22]. The Cronbach’s α coefficient of SF-36 scale in this study was 0.882, and the Cronbach’s α coefficient of Physical health and Mental health aspects of SF-36 scale were 0.826 and 0.869, respectively.

### Other variables

Other variables in this survey included the self-designed social-demographics questions, such as age group, gender, educational background, course of hypertension, complications, family history of hypertension, and types of antihypertensive drugs. The Bai ethnic elders were divided into five age groups (60–64, 65–69, 70–74, 75–79, and 80 years and older) based on our past meaningful findings. Education background was classified into four categories: illiterate, primary school, junior high school, and senior high school and above. There were three categories of course of hypertension, including less than five, five to ten, and ten and above. There were three categories of types of antihypertensive drugs, including one, two, three, and above. “Yes” or “no” choices were given to the participants to answer whether they had complications and a family history of hypertension.

### Statistical analysis

Epidata 3.1 was used to establish the database and Statistical Package for Social Sciences (SPSS) version 22.0 was used to analyze the collected data. Descriptive statistics such as means and standard deviations were utilized to express the statistical results of continuous variables, while proportions and frequencies were applied to show categorical variables. ANOVA was used to examine the statistical differences of variables among different age groups. Pearson correlation analysis was used to analyze the associations among SF-36 and HPLP-II, ESCA in the sampling population. Multiple linear regression analysis was utilized to assess the associations of HPLP-II, ESCA, and other socio-demographic variables on SF-36, by utilizing the total score of SF-36 as a dependent variable. A *P* value of less than 0.05 was considered statistical significance.

## Results

### Baseline sociodemographic and clinical characteristics of participants

A total of 472 Bai ethnic older patients with hypertension were enrolled in this research and responded to the interviews, representing an overall response rate of 99.8%. There were 294 females (62.3%) and 178 males (37.7%), and the mean age of this population was (70.50 ± 7.21) years old. There was no significant difference in gender composition among the different age groups (*P*> 0.05). Moreover, the mean systolic blood pressure of the subjects was 140.82 ± 19.25 mmHg, and the mean diastolic blood pressure of them was 83.03 ± 13.18 mmHg. Among them, 40.0% of the enrolled participants received primary education, and 70.1% of the respondents were married. Approximately 85.8% of the participants had a hypertension course of fewer than 5 years, and 17.4% of the patients had complications,17.8% of them had a hypertension family history, 71.6% of them received 1 type of antihypertensive drug. The social demographic, as well as clinical characteristics of the participating subjects, are illustrated in Table 1.

**Table 1:** Socio-demographic characteristics of the study population (*n* = 472)

Domain	Number (n)	Percent(%)
Gender		
Male	178	37.7
Female	294	62.3
Age group (year)		
60–64	116	24.6
65–69	117	24.8
70–74	97	20.6
75–79	78	16.5
80 and above	64	13.6
Education level		
Illiterate	182	38.6
Primary school	189	40.0
Middle school	74	15.7
High school and above	27	5.7
Marital status		
Married	331	70.1
Divorce or bereavement	141	29.9
The Course of hypertension (year)		
< 5	405	85.8
5–10	28	5.9
≥ 10	39	8.3
Complication		
Yes	82	17.4
No	390	82.6
Family history of hypertension		
Yes	84	17.8
No	388	82.2
Types of antihypertensive drugs		
1	338	71.6
2	108	22.9
≥3	26	5.5

### Comparison of HPLP-II and ESCA scores among different age groups

The scores of HPLP and ESCA of the study population were consistent with the normal distribution, and the scores of HPLP and ESCA among different age groups were demonstrated in Table 2. The average HPLP-II and ESCA scores in this population were calculated as (118.30 ± 19.99) and (106.12 ± 19.47), respectively. The percentage of poor, general and good health-promoting lifestyle level of Bai minority older hypertension patients was 10.2%, 60.6%, and 29.2% respectively. The percentage of the low, medium, and high levels of self-care ability of them was 36.9%, 59.7%, and 3.4%, respectively, which suggests that the health-promoting lifestyle level and self-care ability of the subjects of Bai minority older hypertension patients were not ideal, and the mean HPLP-II and ESCA scores had no significantly different among different age groups (all *P*> 0.05).

**Table 2:** One-way ANOVA analysis of HPLP-II and ESCA scores among different age groups

Age group (year)	HPLP-II (mean ± SD)	ESCA (mean ± SD)
60–64	118.26 ± 21.04	107.61 ± 20.23
65–69	121.78 ± 18.22	108.74 ± 16.62
70–74	117.76 ± 21.99	104.43 ± 20.15
75–79	115.36 ± 19.59	102.85 ± 21.04
80 and above	116.42 ± 17.99	105.17 ± 19.60
Total	118.30 ± 19.99	106.12 ± 19.47
*P* value	0.209	0.208

### Comparison of eight domains and total scores of SF-36 scale among different age groups

The total scores of SF-36 of the study population was consistent with the normal distribution, and eight domains and total scores of SF-36 among all age groups were revealed in Table 3. The mean total score in this Bai minority older hypertension patients of SF-36 was (65.05 ± 13.84), and the scores of physical health and mental health were (72.90 ± 19.90) and (49.55 ± 18.71), which meant the health-related quality of life among the sampling population were poor. The highest score was PF (83.56 ± 17.28), followed by BP (75.66 ± 18.08), RE (68.57 ± 42.66), RP (68.38 ± 44.22), MH (58.57 ± 11.77), GH (57.35 ± 17.67), VT (55.82 ± 13.45), and SF (52.49 ± 17.41). These results indicated that the quality of life was not favorable in Bai minority older hypertension patients in Yunnan province. Statistical differences were found in Bai’s older subjects in the domain of PF and Physical health as well as the overall score in SF-36(all *P*< 0.01), and the 60–64 year group had the highest score in both PF and overall score in SF-36 than other age groups.

**Table 3:** Comparison of eight domains and total score of SF-36 scale between Bai minority elderly in Yunnan province (mean ± SD)

Domain	60–64 year group	65-69year group	70-74year group	75-79year group	80 and aboveyear group	*P* value	Total sample
PF	91.55 ± 12.50	86.88 ± 14.48	83.71 ± 15.04	78.85 ± 18.95	68.52 ± 19.57 ^a^	< 0.001	83.56 ± 17.28
RP	70.69 ± 43.02	72.44 ± 41.44	69.85 ± 44.19	62.82 ± 46.60	61.33 ± 48.17	0.372	68.38 ± 44.22
BP	77.59 ± 17.31	74.04 ± 17.20	77.35 ± 18.23	72.98 ± 18.62	75.86 ± 19.97	0.307	75.66 ± 18.08
GH	60.38 ± 17.00	57.31 ± 18.05	58.32 ± 17.40	54.28 ± 17.55	54.17 ± 18.09	0.086	57.35 ± 17.67
VT	54.87 ± 13.96	55.51 ± 12.52	57.99 ± 12.56	56.54 ± 14.17	53.91 ± 14.46	0.322	55.82 ± 13.45
SF	55.50 ± 17.31	52.24 ± 16.55	53.74 ± 17.13	50.00 ± 18.68	48.63 ± 17.27	0.068	52.49 ± 17.41
RE	72.70 ± 38.72	69.80 ± 40.59	67.35 ± 44.35	67.52 ± 45.58	61.98 ± 47.09	0.587	68.57 ± 42.66
MH	57.97 ± 11.33	60.21 ± 12.86	60.04 ± 11.23	57.79 ± 11.23	55.38 ± 11.41	0.057	58.57 ± 11.77
Physical health	77.75 ± 19.78	74.60 ± 17.66	74.21 ± 18.61	67.74 ± 20.22	65.26 ± 22.38 ^a^	< 0.001	72.90 ± 19.90
Mental health	49.28 ± 16.37	49.63 ± 20.18	50.19 ± 19.59	50.30 ± 18.74	48.01 ± 18.94	0.952	49.55 ± 18.71
Total score of SF-36 scale	67.65 ± 12.22	66.05 ± 13.24	66.04 ± 14.20	62.60 ± 14.59	59.97 ± 14.80 ^a^	0.002	65.05 ± 13.84

^a^denotes statistical significance compared with other age groups

### Correlation analysis between SF-36 and HPLP-II, ESCA

Pearson correlation analysis was utilized to analyze the associations among SF-36 scores and HPLP-II, and ESCA, respectively. Table 4 demonstrated that SF-36 was positively correlated with HPLP-II and ESCA (both *P* < 0.05), which meant that the higher HPLP-II and ESCA levels, the better their health-related quality of life were. And the HPLP-II was positively correlated with ESCA as well (*r* = 0.624, *P*< 0.001).

**Table 4:** Correlation analysis between SF-36 scale scores and HPLP-II, ESCA scale

Domain	PF	RP	BP	GH	VT	SF	RE	MH	Physical health	Mental health	The Total score of SF-36 scale
HPLP-II	0.102^b^	0.214^a^	0.146^a^	0.271^a^	0.055	0.129^a^	0.146^a^	0.018	0.243^a^	0.071	0.240^a^
SECA	0.210^a^	0.193^a^	0.233^a^	0.317^a^	0.242^a^	0.029	0.103^b^	0.135^a^	0.336^a^	0.115^b^	0.190^a^

^a^*P *< 0.01, ^b^*P *< 0.05

## Discussion

In this study, we explored the associations among health-promoting lifestyle, self-care ability, and health-related quality of life in Bai ethnic older patients with hypertension in Yunnan Province. The results of the present study showed that the health promoting lifestyle level and self-care ability of the Bai minority older hypertension patients were low though they had a high awareness rate. There was no difference between HPLP-II and ESCA scores among different age groups in this study. The findings in the total scores of the SF-36 scale indicated that the health-related quality of life in Bai ethnic older subjects was poor according to the ranging standard of Chinese researcher Lei Zhang [23]. Furthermore, the SF-36 scores were positively correlated with HPLP-II and ESCA grades in this sampling population.

Self-care composes the actions that individuals live to lead a healthy lifestyle, take care of their chronic diseases, to prevent further illness [24, 25]. Self-care practice is essential for blood control and the reduction of hypertension complications such as cardiovascular and renal diseases in studies [26]. Hypertension self-care involves dietary and stress management, medication adherence, daily blood pressure monitoring, lifestyle modification, regular healthcare follow-up, etc. Hypertension self-care practice may be affected by a multitude of factors such as demographic factors (age, education, employment, health literacy, illness duration, etc.), self-care agency and self-care efficacy, hypertension knowledge, and social support [25, 26]. The majority of subjects of this study reached an older age and attained hypertension courses for more than 5 years. Furthermore, the vast majority of the Bai hypertension subjects had a primary school education and below, which indicates their education level was very limited. The Bai older subjects in this study could not adhere to the low fat, low salt diet, furthermore, they were found unaccustomed to have milk and fruit in the daily recipes, could not read the labels on the food packages. In addition, the Bai older adults did not realize the importance of monitoring blood pressure, medication adherence, as well as physical exercises in controlling their hypertension. Among of those self-care behaviors, the most prominent self-care problems were the daily monitoring blood pressure, taking antihypertensive medication, regular follow-ups which showed very low adherence levels. That is the reason that could explain why the self-care abilities, health-promoting lifestyles, and HRQOL levels were poor in the Bai subjects. Our study was consistent with some previous research, in which they also reported older age and education are the associated factors with hypertension [14, 15, 27].

HPLP-II involves six aspects of health promotion lifestyles which were the main strategies for health maintenance [19]. These contents of six domains are closely connected with hypertension daily self-care activities. So we found the HPLP-II grades were positively correlated with their ESCA and HRQOL levels, which meant the higher scores of the HPLP-II scales, the better the self-care abilities and health-related quality of life are available to the older adults. This finding was consistent with the study of Zheng et al. [28] which also implied that better HPLP-II was associated with higher QOL. Therefore, it is important to educate the sampling population to adhere to the recommended lifestyle to help them control their blood pressure and attain better HRQOL.

HRQOL is an assessment of health status based on the modern concept of healthcare, which inflects the social, emotional, physical, and psychological well-being of patients [29]. HRQOL is also an essential indicator for hypertension self-care practices. Some researchers reported that good HRQOL would contribute better HPLP-II especially mental health was associated with behaviors, such as self-disciplined exercises, appropriate nutrition, and self-realization [30]. Zhang et al. [31] implied that the influential factor of HRQOL for hypertension patients in China included age, marital status, education level, employment conditions, physical exercises, and regular medical examinations. The Bai hypertensive older adults did not adapt to the healthy lifestyle and regular follow-up check-ups in this study, which contributed to the lower level of their HRQOL together with older age and poor education. Controlled blood pressure was also an essential factor correlated with better health-related quality of life in our study. Controlled blood pressure of Bai older adults reported a more disciplined lifestyle such as restricting sodium, and fat, regular exercise, and follow-up check-ups than their counterparts.

### Implications for practice and research

Some researchers confirmed that social support played a critical role in affecting HPLP-II and QOL [32]. One of the crucial social support measures for hypertension patients is regular healthcare examination. Periodical healthcare follow-up is an essential component of self-care interventions for hypertension patients. It can not only improve the lifestyle, monitoring blood pressure, adherence to the medication for hypertension but also facilitate early detection and treatment of complications with hypertension [33]. Ozoemena et al. [34] suggested that community-based health education intervention targeted at older hypertensive adults can increase hypertension self-care knowledge and improve self-care practices at the population level. Hence, it is vital to publicize the importance of health examinations and then enhance their health awareness of hypertension treatment. Our study proposes local healthcare centers pay more attention to hypertension health education and regular follow-up for Bai ethnic older adults.

### Study limitations

This study has several limitations. Firstly, this cross-sectional design does not allow inferring causality, so it is crucial to follow up with a longitudinal study. Secondly, self-reported indicators were used to collect data in this research, which may induce recall bias regarding the accurate indicators.

## Conclusion

This study explored the associations among the health-promoting lifestyle, self-care agency, and health-related quality of life in Bai ethnic older patients with hypertension in Yunnan Province. The results revealed that the HPLP-II, ESCA, and HRQOL levels were poor among the Bai older subjects. Moreover, their HRQOL scores were positively correlated with HPLP-II and ESCA grades. More social supports targeting health education and regular follow-up check-ups should be paid for by the local healthcare center and government. Meanwhile, lifestyle modification is as important as monthly medical examinations for the sampling population.

## Data Availability

The datasets used and/or analyzed during the current study are available from the corresponding author on reasonable request.

## Abbreviations

HPLP-II-II: Health-promoting lifestyle profile II
ESCAS: Exercise of Self-Care Agency Scale
HRQOL: health-related quality of life
SF-36: MOS 36-Item Short Form Health Survey
